# Demonstrating the non-inferiority of robotic radical cystectomy for cT3–cT4 urothelial carcinoma in the era of neoadjuvant chemotherapy: a propensity score-matched analysis

**DOI:** 10.1007/s11701-025-02771-x

**Published:** 2025-09-12

**Authors:** Il Woo Park, Tae Il Noh, Seok Ho Kang, Jong Jin Oh, Seung Hwan Jeong, Won Sik Ham, Jieun Heo, Hyun Hwan Sung, Byong Chang Jeong, Geehyun Song, Ho Kyung Seo, Kyung Hwan Kim, Jong Kil Nam, Wook Nam, Yun-Sok Ha, Joongwon Choi, Wan Song, Bumjin Lim

**Affiliations:** 1 Department of Urology, Asan Medical Center, Ulsan University College of Medicine, 88 Olympic-Ro 43-Gil, Songpa-Gu, Seoul, 05505 Republic of Korea; 2 Department of Urology, Anam Hospital, Korea University College of Medicine, 73 Inchon-Ro, Seongbuk-Gu, Seoul, 02841 Republic of Korea; 3 Department of Urology, Seoul National University Bundang Hospital, 82 Gumi-Ro 173Beon-Gil, Bundang-Gu, Seongnam-Si, Gyeonggi-Do 13620 Republic of Korea; 4 Department of Urology, Seoul National University Hospital, 101 Daehak-Ro, Jongno-Gu, Seoul, 03080 Republic of Korea; 5 Department of Urology and Urological Science Institute, Yonsei University College of Medicine, 50-1 Yonsei-Ro, Seodaemun-Gu, Seoul, 03722 Republic of Korea; 6 Department of Urology, Samsung Medical Center, Sungkyunkwan University School of Medicine, 81 Irwon-Ro, Gangnam-Gu, Seoul, 06351 Republic of Korea; 7 Department of Urology, Center for Urologic Cancer, National Cancer Center, 323 Ilsan-Ro, Ilsandong-Gu, Goyang-Si, Gyeonggi-Do 10408 Republic of Korea; 8 Department of Urology, School of Medicine, Pusan National University Hospital, Pusan National University, 179 Gudeok-Ro, Seo-Gu, Busan, 49241 Republic of Korea; 9 Department of Urology, School of Medicine, Pusan National University Yangsan Hospital, Pusan National University, 20 Geumo-Ro, Mulgeum-Eup, Yangsan-Si, Gyeongsangnam-Do 50612 Republic of Korea; 10 Department of Urology, Gangneung Asan Hospital, University of Ulsan College of Medicine, 38 Bangdong-Gil, Sacheon-Myeon, Gangneung-Si, Gangwon-Do 25440 Republic of Korea; 11 Department of Urology, Kyungpook National University School of Medicine, 130 Dongdeok-Ro, Jung-Gu, Daegu, 41944 Republic of Korea; 12 Department of Urology, Chung-Ang University Gwangmyeong Hospital, 120-1 Gwangmyeong-Ro, Gwangmyeong-Si, Gyeonggi-Do 14353 Republic of Korea

**Keywords:** Carcinoma, Transitional Cell, Neoadjuvant Therapy, Cystectomy, Robotics, Propensity Score

## Abstract

We evaluated whether robot-assisted radical cystectomy (RARC) is non-inferior to open radical cystectomy (ORC) in patients with cT3–cT4 urothelial carcinoma receiving cisplatin-based neoadjuvant chemotherapy (NAC). We retrospectively analyzed 204 patients (ORC = 123, RARC = 81) across 11 centers. A 1:1 propensity score matching based on age, sex, T stage, and nodal status minimized the selection bias. Recurrence-free survival (RFS), cancer-specific survival (CSS), and overall survival (OS) were compared using Kaplan–Meier analyses and log-rank tests. Cox regression identified independent prognostic factors. Before PSM, the RARC group included younger patients and had fewer individuals with cT4 tumors. Following PSM, 81 patients remained in each arm with balanced characteristics. RARC and ORC showed similar RFS (log-rank *p* = 0.90) and CSS (*p* = 0.16), whereas OS slightly favored RARC (*p* = 0.049). In a multivariable analysis, the surgical approach did not independently predict oncologic outcomes; instead, advanced pathologic stage (≥ pT2), lymphovascular invasion, and nodal involvement (≥ N1) were significant risk factors. The operative time was longer, but blood loss was lower in RARC, with no significant difference in positive margins or major complications. RARC demonstrated non-inferior oncologic outcomes compared to ORC in patients with cT3–cT4 urothelial carcinoma treated with NAC. These findings support the feasibility of a minimally invasive approach without compromising efficacy in advanced bladder cancer.

## Introduction

Radical cystectomy (RC) has long been considered to be a cornerstone in the management of muscle-invasive bladder cancer (MIBC) and, in certain circumstances, for high-risk non-MIBC refractory to intravesical therapy or inadequately controlled by transurethral resection of bladder tumor (TURBT). The advent of robot-assisted RC (RARC) has broadened the surgical options for bladder cancer, offering a minimally invasive technique that can decrease perioperative morbidity, shorten hospital stays, and reduce blood loss [1–3]. However, its role in locally advanced (cT3–cT4) bladder cancer remains controversial, partly due to the larger tumor burden and deeper invasion associated with such high-stage tumors [4–6]. Critics have questioned whether RARC can reliably achieve negative surgical margins and robust lymph node dissection in more extensive diseases, especially given the limited tactile feedback inherent to a robotic platform [7].

For patients with cT3–cT4 bladder cancer, cisplatin-based neoadjuvant chemotherapy (NAC) is a fundamental component of care. Multiple trials have demonstrated that NAC improves recurrence-free survival (RFS) and cancer-specific survival (CSS) by facilitating tumor downstaging and micrometastatic control [8–10]. On the basis of such evidence, the current guidelines endorse NAC in suitable candidates with advanced urothelial carcinoma [9, 11]. Nonetheless, the selection of the subsequent surgical modality remains a subject of debate. Historically, open radical cystectomy (ORC) has been favored for advanced disease, because direct tactile feedback is perceived to help manage complex pelvic anatomies [12]. Adding to this complexity, the effects of NAC—ranging from tumor shrinkage to changes in tissue planes—can occasionally render surgery more challenging if there is an incomplete response or chemo-associated tissue friability. As surgical expertise has grown and robotic technology has advanced, an increasing body of literature suggests that RARC can be performed safely, even for high-stage cancers [6, 13, 14].

Despite these encouraging reports, the comparative data focusing on patients with cT3–cT4 bladder cancer who undergo NAC before RC remain limited, and several existing studies are confounded by small sample sizes, heterogeneous populations, or a lack of robust statistical adjustment for baseline differences [4, 8]. While some smaller series indicate that RARC might achieve oncologic outcomes comparable to ORC in advanced disease, questions persist regarding the generalizability of these findings to truly aggressive tumors [5, 14]. Concerns over intracorporeal urinary diversion, extended operative times, and rare but significant complications—such as port-site seeding—continue to fuel debate [15, 16]. Moreover, NAC-induced toxicities and pre-existing comorbidities frequently complicate perioperative management, necessitating greater clarity on the surgical safety and postoperative recovery in this subset. Finally, extensive data on how each surgical approach affects the timing of additional therapies (such as consolidation systemic therapy), along with functional and quality-of-life endpoints, remain essential to inform optimal treatment pathways [17, 18]. This study aimed to compare the oncologic outcomes of RARC vs. ORC in patients with cT3–cT4 urothelial carcinoma who received cisplatin-based NAC [5].

## Patients and methods

### Patient population

Between January 2010 and December 2019, we retrospectively assembled data on 3258 patients who underwent RC for MIBC at 11 major urological centers throughout Korea. In June 2024, following an initiative by the Bladder Cancer Research Group of the Korean Urological Oncology Society, a web-based database was established, and a single dedicated manager collected and verified all information. The included patients presented with a range of initial TURBT stages—Ta (non-invasive papillary carcinoma), T1 (tumor invading the subepithelial connective tissue), T2 (tumor invading the muscle layer), and Cis (carcinoma in situ). The data encompassing demographic details, tumor characteristics, and therapeutic interventions, such as NAC, final pathology, and lymph node dissection, were meticulously gathered for analysis. The present study was conducted with the approval and oversight of the institutional review board of Asan Medical Center, Seoul, Korea (IRB approval number 2025–0543).

Because of its retrospective nature, the need for informed consent was waived. Patients who had radiologically or clinically proven metastatic disease, previous pelvic radiotherapy, major comorbid conditions precluding surgery, or metallic implants that contraindicated robot-assisted surgery were excluded. All included patients underwent cisplatin-based NAC, classified into gemcitabine–cisplatin, cisplatin–methotrexate–vinblastine, or dose-dense methotrexate–vinblastine–doxorubicin–cisplatin, based on clinician judgment and patient tolerance. The baseline data included demographic factors, such as age, sex, body mass index (BMI), clinical staging (cT and cN), and relevant comorbidities (hypertension, diabetes), as well as the American Society of Anesthesiologists (ASA) classification.

Clinical staging of cT3–T4 disease was determined by radiological evaluation, including chest and abdominopelvic CT in all patients. Pelvic MRI was additionally performed in selected cases at the discretion of the attending physician or when non-contrast imaging was required due to impaired renal function. The staging methodology was consistent across institutions and did not differ between patients treated with ORC and those treated with RARC.

### Surgical procedures

RC was performed using either the ORC or RARC approach. Urinary diversion (ileal conduit or orthotopic neobladder) was selected according to patient preference and preoperative evaluation. For the open approach, a midline laparotomy was used for pelvic dissection and reconstruction. In the robotic group, ports were placed following a standard or extended configuration, and pelvic lymph node dissection (PLND) was performed with robotic instruments; bowel work and urinary diversion were performed extracorporeally. Conversion from robotic to open surgery was allowed if technical or anatomical difficulties occurred. The operative time, estimated blood loss (EBL), and surgical details were recorded.

All RARC procedures in this study were performed by experienced robotic surgeons. Across the 11 participating institutions, a total of 887 RARC cases were performed between 2010 and 2019. Each participating surgeon had previously performed more than 200 cases of robot-assisted laparoscopic prostatectomy (RALP). In Korea, RARC is generally regarded as one of the most technically demanding robotic procedures; therefore, in most centers, RARC was predominantly performed by surgeons with substantial prior robotic experience.

### Follow-up

Postoperative surveillance included routine physical examinations, imaging (chest and abdominopelvic computed tomography), and urine cytology every 3–4 months for the first 2 years, then biannually until 5 years, and annually thereafter. Recurrence was defined as either local (pelvis or cystectomy bed) or distant (extrapelvic nodes or visceral metastases) on radiological or histopathological assessment. Perioperative outcomes (such as complications) and pathologic findings (lymph node yield, margin status) were extracted from the medical records.

### Statistical analysis

Initially, all patients were categorized into either the ORC group or RARC group. The baseline characteristics were compared using Pearson’s chi-square test or Fisher’s exact test for categorical variables and Student’s *t* test or Wilcoxon rank-sum test for continuous variables, as appropriate. RFS, CSS, and overall survival (OS) were estimated using the Kaplan–Meier (KM) method and differences between groups were assessed using the log-rank test. Independent predictors of RFS, CSS, and OS were identified with Cox proportional hazards regression, including relevant covariates.

To correct for potential selection bias between the ORC and RARC groups, a 1:1 propensity score matching (PSM) was performed. The logistic regression model for generating the propensity score included age, sex, clinical T stage, and clinical node status as covariates. Matching quality was evaluated by standardized mean differences, with values below 10% indicating acceptable balance. After matching, KM curves were reconstructed for each endpoint. All statistical tests were two-sided, and *p* < 0.05 was considered statistically significant. All analyses were conducted using R version 4.4.2.

## Results

### Patient characteristics and baseline comparisons

The study population comprised patients with cT3–cT4 urothelial carcinoma who had received NAC. Of these, 123 patients underwent ORC, and 81 had RARC. Before PSM, there were notable differences between the two cohorts in median age, BMI, ASA classification, and clinical T stage (Table 1).

**Table 1:** Baseline characteristics before propensity score matching

Variable	Category	Open	Robot	*p* value
Age		75.0 (69.0–81.0)	70.0 (64.0–77.0)	0.003
Sex	Male	108 (87.8%)	71 (87.7%)	1.000
	Female	15 (12.2%)	10 (12.3%)	
BMI		23.6 (21.1–25.2)	24.3 (22.5–26.1)	0.023
ASA	ASA1	15 (12.2%)	1 (1.2%)	0.010
	2up	108 (87.8%)	80 (98.8%)	
HTN	No	75 (61.0%)	51 (63.0%)	0.890
	Yes	48 (39.0%)	30 (37.0%)	
DM	No	95 (77.2%)	58 (71.6%)	0.457
	Yes	28 (22.8%)	23 (28.4%)	
LVI	No	70 (56.9%)	63 (77.8%)	0.004
	Yes	53 (43.1%)	18 (22.2%)	
CIS_positive	No	93 (75.6%)	57 (70.4%)	0.504
	Yes	30 (24.4%)	24 (29.6%)	
LN_invasion	No	86 (69.9%)	62 (76.5%)	0.380
	Yes	37 (30.1%)	19 (23.5%)	
Histology_divided	Urothelial	98 (79.7%)	69 (85.2%)	0.416
	Variant	25 (20.3%)	12 (14.8%)	
Concurrent_UTUC	None	113 (91.9%)	78 (96.3%)	0.497
	Unilat	5 (4.1%)	2 (2.5%)	
	Bilat	5 (4.1%)	1 (1.2%)	
RCx_with_NUx	No	113 (91.9%)	79 (97.5%)	0.130
	Yes	10 (8.1%)	2 (2.5%)	
Clinical_T_stage	T3	89 (72.4%)	71 (87.7%)	0.015
	T4	34 (27.6%)	10 (12.3%)	
Clinical_N_stage	N0	76 (61.8%)	64 (79.0%)	0.056
	N1	21 (17.1%)	10 (12.3%)	
	N2	22 (17.9%)	6 (7.4%)	
	N3	4 (3.3%)	1 (1.2%)	
Operation_time_min		366.0 (98.6)	426.7 (142.7)	0.001
EBL_ml		650.0 (400.0–1300.0)	300.0 (300.0–600.0)	< 0.001
Diversion_type	Ileal conduit	74 (60.2%)	28 (34.6%)	< 0.001
	Orthotopic neobladder	45 (36.6%)	53 (65.4%)	
	Ureterocutaneostomy	4 (3.3%)	0 (0.0%)	
PLND_type	None	6 (4.9%)	3 (3.7%)	0.151
	Limited	6 (4.9%)	3 (3.7%)	
	Standard	75 (61.0%)	57 (70.4%)	
	Extended	28 (22.8%)	18 (22.2%)	
	Super_extended	8 (6.5%)	0 (0.0%)	
Pathologic_T_stage	T2 < =	46 (37.4%)	45 (55.6%)	0.005
	T3	51 (41.5%)	28 (34.6%)	
	T4	26 (21.1%)	8 (9.9%)	
Pathologic_N_stage	N0	77 (62.6%)	59 (72.8%)	0.316
	N1	19 (15.4%)	9 (11.1%)	
	N2plus	27 (22.0%)	13 (16.0%)	
Corporeal_type	Open	123 (100.0%)	0 (0.0%)	< 0.001
	Robot_Extra	0 (0.0%)	40 (49.4%)	
	Robot_intra	0 (0.0%)	41 (50.6%)	
Recurrence	No	72 (58.5%)	48 (59.3%)	1.000
	Yes	51 (41.5%)	33 (40.7%)	
Positive_surgical_margin	No	104 (84.6%)	77 (95.1%)	0.036
	Yes	19 (15.4%)	4 (4.9%)	
Neo_adjuvant_regimen	GP	109 (88.6%)	76 (93.8%)	0.372
	CMV	1 (0.8%)	0 (0.0%)	
	ddMVAC	13 (10.6%)	5 (6.2%)	

*BMI* body mass index, *ASA* American Society of Anesthesiologists Physical Status Classification, *HTN* hypertension, *DM* diabetes mellitus, *LVI* lymphovascular invasion, *CIS* carcinoma in situ, *LN* lymph node, *UTUC* upper tract urothelial carcinoma, *EBL* estimated blood loss, *PLND* pelvic lymph node dissection, *GP* gemcitabine–cisplatin, *CMV* cisplatin–methotrexate–vinblastine, *ddMVAC* dose-dense methotrexate–vinblastine–doxorubicin–cisplatin.

Specifically, patients in the open group were generally older (median age: 75 vs. 70 years, *p* < 0.01) and had a lower BMI (23.6 vs. 24.3 kg/m^2^, *p* < 0.05). Moreover, ASA ≥ 2 status was more prevalent in the robotic group (*p* = 0.01), and a higher proportion of cT4 tumors was observed in the open surgery cohort (*p* < 0.05).

The median operative time was significantly longer in the RARC group (420 min) than in the ORC group (363 min, *p* < 0.01). Conversely, median EBL was lower in the RARC cohort (300 mL) vs. the open cohort (600 mL, *p* < 0.01), suggesting a potential surgical advantage of minimally invasive techniques. The proportion of patients receiving orthotopic neobladder was higher in the RARC arm (65% vs. 44%, *p* < 0.05). Postoperative margin positivity showed a trend favoring the robotic group (4.9% vs. 14.8%, *p* = 0.065 post-match); however, the difference was not statistically significant. The PLND type (standard or extended) was balanced between groups, and no major discrepancies in pathologic T or N staging distributions remained after matching.

### Survival outcomes

**Fig. 1:**
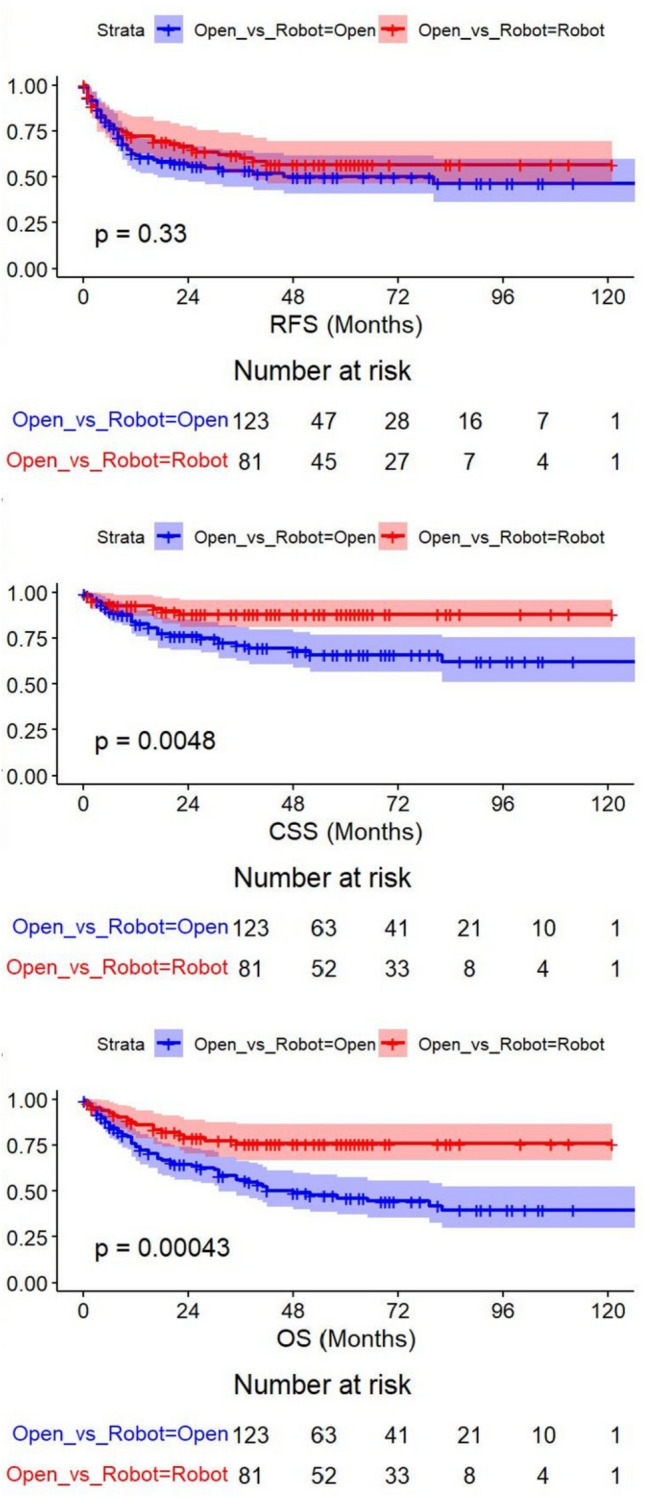
Kaplan–Meier curves for recurrence-free survival (RFS), cancer-specific survival (CSS), and overall survival (OS) before PSM

In univariable analyses conducted before PSM, RARC was associated with significantly improved CSS (*p* = 0.0048) and OS (*p* = 0.00043), whereas RFS showed no significant difference (*p* = 0.33) (Fig. 1).

However, once key covariates were accounted for in a stepwise multivariate model, the surgical approach (RARC vs. ORC) lost its statistical significance (*p* > 0.05) and was excluded from the final model. In contrast, advanced pathologic T stage (≥ T2), lymphovascular invasion (LVI), and nodal disease (≥ N1) consistently emerged as adverse prognostic factors (Table 2).

**Table 2:** Univariate and multivariate Cox regression analyses of survival outcomes

	RFS: recurrence-free survival			
Characteristics	Univariate		Multivariate	
	HR (95% CI)	*p* value	HR (95% CI)	*p* value
Open_vs_Robot (Robot)	0.80 (0.52–1.25)	0.336	–	–
Age	1.00 (0.98–1.02)	0.977	–	–
Sex (female)	1.58 (0.89–2.80)	0.119	1.84 (1.01–3.34)	0.046
BMI	1.00 (0.94–1.06)	0.918	–	–
ASA (≥ 2)	0.68 (0.33–1.42)	0.309	0.34 (0.15–0.73)	0.006
HTN	1.15 (0.74–1.78)	0.534	–	–
DM	0.78 (0.46–1.31)	0.351	–	–
LVI	3.98 (2.56–6.17)	< 0.001	2.72 (1.59–4.63)	< 0.001
CIS	0.91 (0.56–1.47)	0.691	–	–
PLND	0.40 (0.17–0.92)	0.032	0.28 (0.11–0.72)	0.008
Concurrent_UTUC	1.70 (0.78–3.70)	0.180	–	–
Clinical_T_stage (T4)	2.43 (1.54–3.84)	< 0.001	2.23 (1.38–3.62)	0.001
Diversion_type (ONB)	0.56 (0.36–0.87)	0.010	–	–
Pathologic_T_stage (≥ T2)	4.32 (2.23–8.38)	< 0.001	2.27 (1.07–4.82)	0.033
Pathologic_N_stage (≥ N1)	4.25 (2.74–6.58)	< 0.001	2.11 (1.24–3.60)	0.006
LN_invasion	3.84 (2.48–5.92)	< 0.001	–	–
Histology (Other)	1.52 (0.90–2.56)	0.336	–	–
Positive_surgical_margin	3.27 (1.91–5.59)	0.977	–	–

	CSS: cancer-specific survival;			
Characteristics	Univariate		Multivariate	
	HR (95% CI)	*p* value	HR (95% CI)	*p* value
Open_vs_Robot (Robot)	0.36 (0.17–0.76)	0.007	–	–
Age	1.00 (0.97–1.03)	0.895	–	–
Sex (female)	1.03 (0.40–2.63)	0.949	–	–
BMI	0.95 (0.86–1.04)	0.256	–	–
ASA (≥ 2)	0.84 (0.30–2.35)	0.736	–	–
HTN	1.08 (0.58–2.01)	0.810	–	–
DM	1.31 (0.68–2.52)	0.421	–	–
LVI	2.62 (1.42–4.82)	0.002	–	–
CIS	1.22 (0.63–2.35)	0.549	–	–
PLND	0.28 (0.10–0.78)	0.015	–	–
Concurrent_UTUC	1.29 (0.40–4.18)	0.674	–	–
Clinical_T_stage (T4)	2.75 (1.47–5.13)	0.002	2.43 (1.27–4.65)	0.007
Diversion_type (ONB)	0.50 (0.27–0.94)	0.033	–	–
Pathologic_T_stage (≥ T2)	4.87 (1.73–13.66)	0.003	3.72 (1.26–10.98)	0.017
Pathologic_N_stage (≥ N1)	3.21 (1.74–5.92)	< 0.001	6.53 (2.45–17.45)	< 0.001
LN_invasion	2.18 (1.17–4.04)	0.014	0.26 (0.10–0.70)	0.008
Histology (Other)	2.05 (1.05–4.01)	0.036	–	–
Positive_surgical_margin	3.75 (1.88–7.48)	< 0.001	–	–

	OS: overall survival			
Characteristics	Univariate		Multivariate	
	HR (95% CI)	*p* value	HR (95% CI)	*p* value
Open_vs_Robot (Robot)	0.40 (0.24–0.68)	< 0.001	–	–
Age	1.03 (1.00–1.05)	0.041	–	–
Sex (female)	1.71 (0.96–3.06)	0.069	–	–
BMI	0.96 (0.89–1.03)	0.206	–	–
ASA (≥ 2)	0.88 (0.40–1.91)	0.739	–	–
HTN	1.32 (0.84–2.08)	0.227	–	–
DM	1.08 (0.66–1.79)	0.755	–	–
LVI	2.70 (1.72–4.24)	< 0.001	–	–
CIS	0.95 (0.57–1.59)	0.855	–	–
PLND	0.40 (0.16–0.98)	0.046	–	–
Concurrent_UTUC	1.19 (0.48–2.96)	0.704	–	–
Clinical_T_stage (T4)	1.96 (1.20–3.19)	0.007	–	–
Diversion_type (ONB)	0.35 (0.22–0.58)	< 0.001	0.43 (0.26–0.71)	0.001
Pathologic_T_stage (≥ T2)	3.57 (1.83–6.95)	< 0.001	2.18 (1.05–4.53)	0.037
Pathologic_N_stage (≥ N1)	2.99 (1.91–4.70)	< 0.001	4.07 (1.83–9.04)	< 0.001
LN_invasion	2.37 (1.50–3.73)	< 0.001	0.35 (0.15–0.80)	0.013
Histology (Other)	1.46 (0.85–2.51)	0.168	–	–
Positive_surgical_margin	2.41 (1.35–4.30)	0.003	–	–

*HR* hazard ratio, *CI* confidence interval, *BMI* body mass index, *ASA* American Society of Anesthesiologists Physical Status Classification, *HTN* hypertension, *DM* diabetes mellitus, *LVI* lymphovascular invasion, *CIS* carcinoma in situ, *PLND* pelvic lymph node dissection, *UTUC* upper tract urothelial carcinoma, *ONB* orthotopic neobladder, *LN* lymph node.

RARC showed a distinct advantage over ORC for CSS and OS, but not for RFS. After PSM, however, the difference in OS remained only borderline significant in favor of RARC (*p* = 0.049), whereas CSS (*p* = 0.16) and RFS (*p* = 0.90) were no longer significantly different between groups. In the matched population’s multivariate analysis, the surgical approach again failed to emerge as an independent predictor, implying that the initial survival advantage observed for RARC in unadjusted analyses was largely attributable to baseline imbalances. Overall, these findings indicate that once confounders are adequately controlled, RARC provides oncologic outcomes broadly comparable to ORC in patients with cT3–cT4 urothelial carcinoma undergoing NAC.

### PSM

PSM achieved a substantial reduction in baseline imbalances (Table 3).

**Table 3:** Baseline characteristics after propensity score matching

Variable	Category	Open	Robot	*p* value
Age		75.0 (67.0–80.0)	70.0 (64.0–77.0)	0.072
BMI		23.9 (21.2–25.0)	24.3 (22.5–26.1)	0.045
Operation_time_min		363.0 (291.0–437.0)	420.0 (315.0–540.0)	0.002
EBL_ml		600.0 (330.0–1450.0)	300.0 (300.0–600.0)	< 0.001
Sex	Male	71 (87.7%)	71 (87.7%)	1.000
	Female	10 (12.3%)	10 (12.3%)	
ASA_divided	1	11 (13.6%)	1 (1.2%)	0.007
	2 > =	70 (86.4%)	80 (98.8%)	
Clinical_T_stage	T3	69 (85.2%)	71 (87.7%)	0.819
	T4	12 (14.8%)	10 (12.3%)	
Lymph_node_metastasis	No	63 (77.8%)	64 (79.0%)	1.000
	Yes	18 (22.2%)	17 (21.0%)	
RCx_with_NUx	No	78 (96.3%)	79 (97.5%)	1.000
	Yes	3 (3.7%)	2 (2.5%)	
Diversion_type	IC	41 (50.6%)	28 (34.6%)	0.027
	ONB	36 (44.4%)	53 (65.4%)	
CIS_positive	No	62 (76.5%)	57 (70.4%)	0.477
	Yes	19 (23.5%)	24 (29.6%)	
Histology	Urothelial	67 (82.7%)	69 (85.2%)	0.831
	Variant	14 (17.3%)	12 (14.8%)	
LVI	No	52 (64.2%)	63 (77.8%)	0.083
	Yes	29 (35.8%)	18 (22.2%)	
Positive_surgical_margin	No	69 (85.2%)	77 (95.1%)	0.065
	Yes	12 (14.8%)	4 (4.9%)	
LN_invasion	No	62 (76.5%)	62 (76.5%)	1.000
	Yes	19 (23.5%)	19 (23.5%)	
Recurrence	No	52 (64.2%)	48 (59.3%)	0.628
	Yes	29 (35.8%)	33 (40.7%)	
Clinical_N_stage	N0	63 (77.8%)	64 (79.0%)	0.721
	N1	9 (11.1%)	10 (12.3%)	
	N2	9 (11.1%)	6 (7.4%)	
Concurrent_UTUC	None	78 (96.3%)	78 (96.3%)	1.000
	Unilat	1 (1.2%)	2 (2.5%)	
	Bilat	2 (2.5%)	1 (1.2%)	
Corporeal_type	Open	81 (100.0%)	0 (0.0%)	0.000
	Robot_intra	0 (0.0%)	41 (50.6%)	
	Robot_Extra	0 (0.0%)	40 (49.4%)	
Neo_adjuvant_regimen	GP	69 (85.2%)	76 (93.8%)	0.122
	CMV	1 (1.2%)	0 (0.0%)	
	ddMVAC	11 (13.6%)	5 (6.2%)	
Pathologic_T_stage	T2 < =	37 (45.7%)	45 (55.6%)	0.453
	T3	34 (42.0%)	28 (34.6%)	
	T4	10 (12.3%)	8 (9.9%)	
Pathologic_N_stage	0	58 (71.6%)	59 (72.8%)	0.970
	1	10 (12.3%)	9 (11.1%)	
	2	13 (16.0%)	13 (16.0%)	
PLND_type	None	3 (3.7%)	3 (3.7%)	0.452
	Limited	5 (6.2%)	3 (3.7%)	
	Standard	50 (61.7%)	57 (70.4%)	
	Extended	20 (24.7%)	18 (22.2%)	
	Super_extended	3 (3.7%)	0 (0.0%)	

*BMI* body mass index, *EBL* ASA American Society of Anesthesiologists Physical Status Classification, *CIS* carcinoma in situ, *LVI* lymphovascular invasion, *LN* lymph node, *UTUC* upper tract urothelial carcinoma, *GP* gemcitabine–cisplatin, *CMV* cisplatin–methotrexate–vinblastine, *ddMVAC* dose-dense methotrexate–vinblastine–doxorubicin–cisplatin, *PLND* pelvic lymph node dissection.

**Fig. 2:**
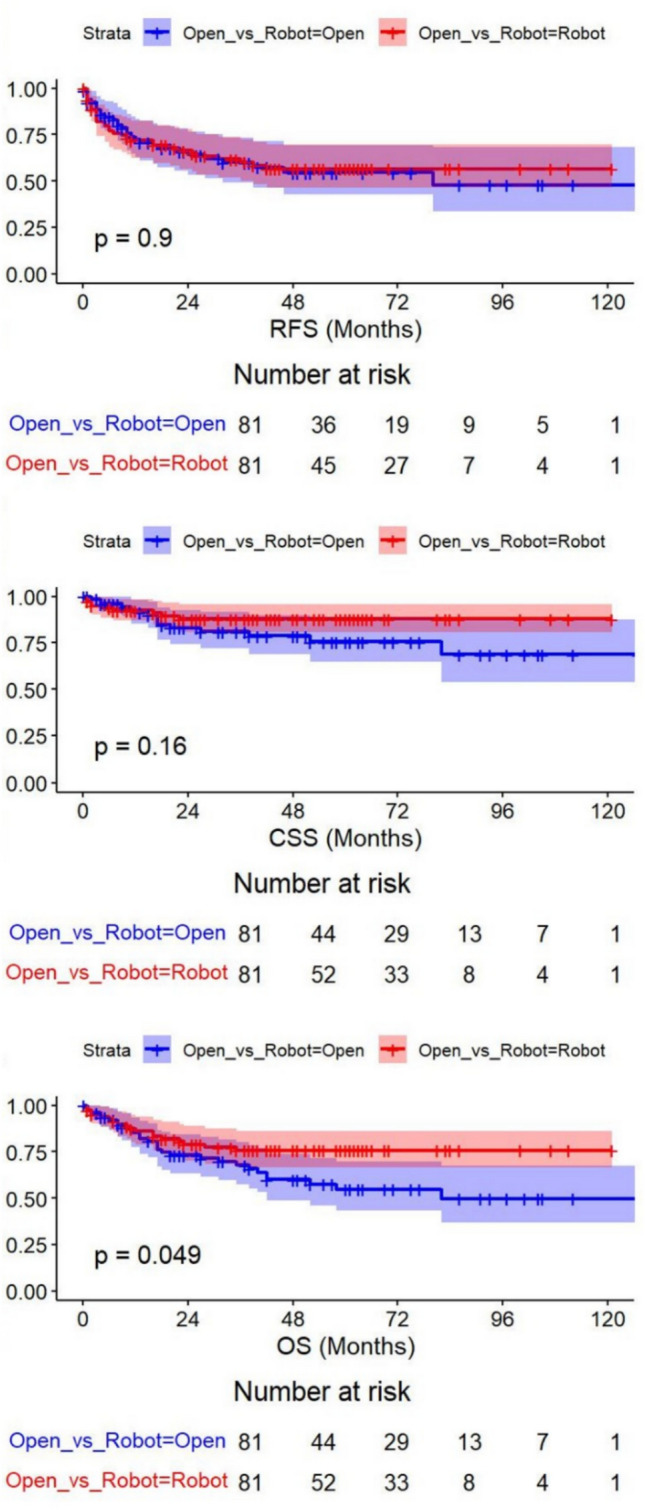
Kaplan–Meier curves for recurrence-free survival (RFS), cancer-specific survival (CSS), and overall survival (OS) after propensity score matching

After matching for age, sex, clinical T stage, and lymph node metastasis, 81 patients were retained in each arm. Post-matching, the standardized mean differences for these key variables decreased to below 10%, indicating an effective balance between cohorts. Although differences in ASA classification and diversion type (ileal conduit vs. orthotopic neobladder) persisted to some degree, no other major covariates were significantly imbalanced. No significant differences were found in age (median age: 73 vs. 70 years, *p* = 0.07), BMI, or clinical T and N distribution. This improvement in distribution was reflected in closely matched baseline standard means (all < 10%), ensuring a robust comparison of surgical approaches. Following PSM (Fig. 2),

the survival curves overlapped even further, indicating no appreciable difference in the oncologic outcomes between ORC and RARC. At 24 months, for example, the RFS estimates in the matched population were similar (approximately 59% for RARC vs. 58% for ORC, log-rank *p* > 0.05), and this pattern persisted at 36 months. Similarly, the 2- and 3-year CSS and OS rates did not differ significantly between groups (all log-rank *p* > 0.05). Overall, these results suggest that once the key confounders are accounted for, RARC achieves oncologic and survival outcomes comparable to ORC in patients with cT3–cT4 urothelial carcinoma undergoing NAC. The log-rank tests further confirmed that survival did not significantly vary by surgical approach for any of the three endpoints.

## Discussion

In this multicenter propensity score-matched analysis of patients with cT3–cT4 urothelial carcinoma who received cisplatin-based NAC, we compared the perioperative outcomes and oncologic efficacy of RARC vs. ORC. Our findings strongly indicate that, after adjusting for key baseline differences, RARC achieves non-inferior RFS, CSS, and OS [6, 19]. This result supports the feasibility of RARC as an alternative to ORC in cases of locally advanced bladder cancer.

Before PSM, the RARC group consisted of younger patients (median age: 70 vs. 75 years, *p* = 0.003) with a lower proportion of cT4 tumors (12.3% vs. 27.6%, *p* = 0.015). Following PSM, these differences were minimized (cT4 proportion: 12.3% vs. 14.8%, *p* = 0.819), enabling a more accurate comparison between the two surgical approaches. Although the ASA distribution was not perfectly balanced, the remaining slight imbalance likely reflects each center’s patient selection policies rather than a bias in the study design [1, 2, 20].

When comparing the perioperative data, two major differences emerged. First, the operative time was significantly longer in the RARC group (median: 420 min vs. 363 min, *p* = 0.0016), which aligns with previous literature suggesting robotic cystectomy can require more time, particularly in complex pelvic situations. However, the EBL was markedly lower for RARC (300 mL vs. 600 mL, *p* < 0.0002), echoing reports that the enhanced visualization and precise dissection facilitated by robotics can reduce surgical bleeding [1, 2, 12, 21]. In addition, a higher proportion of patients underwent orthotopic neobladder reconstruction in the RARC group (65% vs. 44%, *p* = 0.027), possibly reflecting both patient preference and a willingness among surgeons to pursue more complex urinary diversions when employing a minimally invasive approach [3, 15].

Critically, in cT3–cT4 bladder cancer, tactile feedback has historically been considered indispensable for ensuring negative surgical margins and performing extensive lymphadenectomies. However, in our post-matching analysis, margin positivity was numerically lower in the RARC group (4.9% vs. 14.8%, *p* = 0.065), suggesting that, with adequate surgical expertise, even locally advanced disease can be excised effectively via a robotic platform. Although this difference did not reach conventional statistical significance, the trend adds to growing evidence that high-definition visualization and articulated instruments can offset the absence of direct tactile sensation [14, 22, 23].

We focused on patients who received NAC (predominantly gemcitabine–cisplatin), as this approach is recommended for MIBC, particularly cT3–cT4 tumors, to address micrometastases and improve pathologic downstaging. Concerns have been raised that NAC-induced tissue changes or toxicities might compromise surgical safety. However, our results show no significant increase in the perioperative complications attributable to NAC in either the RARC or ORC group, consistent with the notion that NAC need not be a contraindication to minimally invasive approaches [8, 24].

On univariable and multivariable Cox regression, the surgical approach (RARC vs. ORC) did not predict RFS, CSS, or OS (hazard ratio [HR] = 0.80, *p* = 0.336). Instead, the final pathologic features, lymphovascular invasion (LVI), pT stage ≥ 2, and nodal involvement ≥ N1, were the strongest prognostic determinants [6, 18]. KM curves further confirmed overlapping survival patterns between RARC and ORC in both pre- and post-match populations (all *p* > 0.05 by log-rank test). These findings align with previous smaller studies but now offer higher statistical power in a multicenter setting. Despite the complexity associated with cT3–cT4 disease and NAC, we observed no detriment to performing RARC in terms of oncologic endpoints [23, 25].

RC remains a major operation with potentially high morbidity. Importantly, we did not observe significant differences in major complications, readmissions, or 90-day mortality between the two approaches (all *p* > 0.1). While the specific rates of complications in both cohorts ranged from minor wound issues to severe septic events—particularly given the complexity of urinary diversion—the essential insight is that NAC did not amplify complications differently for RARC vs. ORC. This suggests that the potential synergy between systemic chemotherapy and surgical morbidity is manageable when proper patient selection and experienced surgical teams are in place [1, 8, 11].

From a technical standpoint, the most challenging aspect of RARC compared to open surgery is the intracorporeal urinary diversion. The ileum is a highly mobile organ, and within the confined pelvic workspace of robotic surgery, this mobility often hinders visualization and reduces instrument maneuverability. Moreover, the lack of tactile feedback and limited control over grasping force require surgeons to carefully handle the bowel to avoid injury, which complicates the entire process. In our own institutional experience, we converted two cases from robotic to open surgery due to technical difficulties during ileal diversion in patients with dense intra-abdominal adhesions. Critically, however, these technical difficulties and conversions were unrelated to the advanced tumor stage (cT3–T4). This suggests that high-stage disease itself is not a barrier to performing RARC.

Some prior series report 40–50% readmission rates for patients with cystectomy overall, and this study (with approximately 40–45% readmissions in both groups) concurs that the complexity of the procedure, rather than the surgical approach alone, largely drives postoperative challenges [2, 5]. Although robotics may lessen certain morbidities (e.g., transfusions) via reduced EBL, the inherent demands of pelvic lymph node dissection and urinary reconstruction can still lead to significant complication rates, underscoring the importance of robust enhanced recovery after surgery protocols and dedicated postoperative care pathways.

Several clinically relevant points emerge from this study. First, NAC did not adversely affect the feasibility and safety of RARC, reinforcing that advanced local disease in combination with NAC does not mandate an open approach [4, 8]. Second, the reduction in blood loss associated with RARC is a positive finding that highlights the technical refinement of the robotic approach. Third, the higher utilization of orthotopic neobladder in RARC points to the possibility that minimally invasive procedures may be more conducive to complex urinary diversions, although this merits further prospective evaluation [4, 15].

The limitations of this study include its retrospective nature, possible residual confounders, and varying follow-up durations across centers. Although PSM was applied, unknown factors could still influence treatment selection or outcomes. In addition, we lacked a centralized pathology review, and extended analyses of quality-of-life outcomes were not available [21]. Furthermore, our multicenter database did not include data on pre-existing hydronephrosis or baseline eGFR. However, eligibility for cisplatin-based neoadjuvant chemotherapy requires adequate renal function (generally defined as an eGFR ≥ 60 mL/min/1.73 m^2^). Since all patients in our cohort received this regimen, it can be inferred that they had sufficient renal function to tolerate the treatment. Nevertheless, the absence of specific data on hydronephrosis and renal function assessment remains a limitation of this study. In addition, our database did not systematically collect information on the use of postoperative adjuvant therapy [26].

Nevertheless, our multicenter design and a relatively large sample of NAC-treated patients with cT3–cT4 bladder cancer confer a level of real-world generalizability often absent in single-center case series. It should also be noted that while adjuvant therapy was not a standard approach during our study period, recent evidence from the CheckMate 274 trial and subsequent international guideline updates have established adjuvant nivolumab as the standard of care for high-risk patients after cystectomy. This evolving landscape highlights the need for future research to evaluate how novel systemic therapies interact with surgical approaches in advanced bladder cancer [27]. Future research should involve prospective or randomized studies to confirm these conclusions definitively. The role of robotics will likely continue to expand in concert with new systemic therapies—such as checkpoint inhibitors—making the interplay between NAC, immunotherapy, and minimally invasive surgery an important frontier. Cost-effectiveness and long-term functional outcomes (e.g., continence and sexual function) also deserve more rigorous evaluation [6, 19].

In summary, this study strongly supports that RARC is a non-inferior alternative to ORC in the setting of cT3–cT4 urothelial carcinoma post-NAC. The two approaches yielded similar survival outcomes, whereas RARC offered reduced bleeding and no evident increase in mortality or severe complications [6, 20]. These findings endorse a paradigm, where NAC should not deter surgeons from adopting minimally invasive procedures, provided adequate expertise is available. Ultimately, this evidence points toward a future in which RARC is considered a standard option for patients with complex bladder cancer, reinforcing that oncologic safety and patient-centered benefits can align with advanced disease management.

## Conclusions

In cT3–cT4 urothelial carcinoma receiving cisplatin-based NAC, RARC demonstrated RFS, CSS, and OS comparable to ORC after PSM. Although the operative times were longer with RARC, the EBL was significantly lower, and major complications were similar. These findings suggest that a minimally invasive approach remains a viable alternative for advanced bladder cancer without compromising the oncologic outcomes.

## Data Availability

No data sets were generated or analyzed during the current study.

## References

[1] Bochner BH, Dalbagni G, Sjoberg DD et al (2015) Comparing open radical cystectomy and robot-assisted laparoscopic radical cystectomy: a randomized clinical trial. Eur Urol 67(6):1042–1050. 10.1016/j.eururo.2014.11.043 25496767 10.1016/j.eururo.2014.11.043 PMC4424172

[2] Clement KD, Pearce E, Gabr AH, Rai BP, Al-Ansari A, Aboumarzouk OM (2021) Perioperative outcomes and safety of robotic vs open cystectomy: a systematic review and meta-analysis of 12,640 cases. World J Urol 39(6):1733–1746. 10.1007/s00345-020-03385-8 32734460 10.1007/s00345-020-03385-8

[3] Khan MS, Gan C, Ahmed K et al (2016) A single-centre early phase randomised controlled three-arm trial of open, robotic, and laparoscopic radical cystectomy (CORAL). Eur Urol 69(4):613–621. 10.1016/j.eururo.2015.07.038 26272237 10.1016/j.eururo.2015.07.038

[4] Zennami K, Sumitomo M, Takahara K et al (2021) Intra-corporeal robot-assisted versus open radical cystectomy: a propensity score-matched analysis comparing perioperative and long-term survival outcomes and recurrence patterns. Int J Clin Oncol 26(8):1514–1523. 10.1007/s10147-021-01939-3 34009486 10.1007/s10147-021-01939-3

[5] Gandaglia G, Karl A, Novara G et al (2016) Perioperative and oncologic outcomes of robot-assisted vs. open radical cystectomy in bladder cancer patients: a comparison of two high-volume referral centers. Eur J Surg Oncol 42(11):1736–1743. 10.1016/j.ejso.2016.02.254 27032295 10.1016/j.ejso.2016.02.254

[6] Parekh DJ, Reis IM, Castle EP et al (2018) Robot-assisted radical cystectomy versus open radical cystectomy in patients with bladder cancer (RAZOR): an open-label, randomised, phase 3, non-inferiority trial. Lancet 391(10139):2525–2536. 10.1016/S0140-6736(18)30996-6 29976469 10.1016/S0140-6736(18)30996-6

[7] Sharma P, Zargar-Shoshtari K, Poch MA et al (2017) Surgical control and margin status after robotic and open cystectomy in high-risk cases: caution or equivalence? World J Urol 35(4):657–663. 10.1007/s00345-016-1915-2 27495912 10.1007/s00345-016-1915-2

[8] Gandaglia G, Popa I, Abdollah F et al (2014) The effect of neoadjuvant chemotherapy on perioperative outcomes in patients who have bladder cancer treated with radical cystectomy: a population-based study. Eur Urol 66(3):561–568. 10.1016/j.eururo.2014.01.014 24486024 10.1016/j.eururo.2014.01.014

[9] Witjes JA, Bruins HM, Cathomas R et al (2021) European association of urology guidelines on muscle-invasive and metastatic bladder cancer: summary of the 2020 guidelines. Eur Urol 79(1):82–104. 10.1016/j.eururo.2020.03.055 32360052 10.1016/j.eururo.2020.03.055

[10] Pfister C, Gravis G, Flechon A et al (2021) Randomized phase III trial of dose-dense methotrexate, vinblastine, doxorubicin, and cisplatin, or gemcitabine and cisplatin as perioperative chemotherapy for patients with muscle-invasive bladder cancer. Analysis of the GETUG/AFU V05 VESPER trial secondary endpoints: chemotherapy toxicity and pathological responses. Eur Urol 79(2):214–221. 10.1016/j.eururo.2020.08.024 32868138 10.1016/j.eururo.2020.08.024

[11] Chang SS, Bochner BH, Chou R et al (2017) Treatment of non-metastatic muscle-invasive bladder cancer: AUA/ASCO/ASTRO/SUO guideline. J Urol 198(3):552–559. 10.1016/j.juro.2017.04.086 28456635 10.1016/j.juro.2017.04.086 PMC5626446

[12] Bochner BH, Dalbagni G, Marzouk KH et al (2018) Randomized trial comparing open radical cystectomy and robot-assisted laparoscopic radical cystectomy: oncologic outcomes. Eur Urol 74(4):465–471. 10.1016/j.eururo.2018.04.030 29784190 10.1016/j.eururo.2018.04.030 PMC6697266

[13] Hussein AA, Saar M, May PR et al (2017) Early oncologic failure after robot-assisted radical cystectomy: results from the International Robotic Cystectomy Consortium. J Urol 197(6):1427–1436. 10.1016/j.juro.2016.12.048 27993668 10.1016/j.juro.2016.12.048

[14] Iwata T, Kimura S, Foerster B et al (2019) Oncologic outcomes after robot-assisted versus open radical cystectomy: a systematic review and meta-analysis. World J Urol 37(8):1557–1570. 10.1007/s00345-019-02708-8 30976902 10.1007/s00345-019-02708-8

[15] Jonsson MN, Adding LC, Hosseini A et al (2011) Robot-assisted radical cystectomy with intracorporeal urinary diversion in patients with transitional cell carcinoma of the bladder. Eur Urol 60(5):1066–1073. 10.1016/j.eururo.2011.07.035 21852033 10.1016/j.eururo.2011.07.035

[16] Tan WS, Khetrapal P, Tan WP, Rodney S, Chau M, Kelly JD (2016) Robotic assisted radical cystectomy with extracorporeal urinary diversion does not show a benefit over open radical cystectomy: a systematic review and meta-analysis of randomised controlled trials. PLoS ONE 11(11):e0166221. 10.1371/journal.pone.0166221 27820855 10.1371/journal.pone.0166221 PMC5098822

[17] Bharadwaj M, Kaul S, Fleishman A et al (2022) Adjuvant chemotherapy versus observation following radical cystectomy for locally advanced urothelial carcinoma of the bladder. Urol Oncol. 10.1016/j.urolonc.2022.02.002 35307291 10.1016/j.urolonc.2022.02.002

[18] Venkatramani V, Reis IM, Castle EP et al (2020) Predictors of recurrence, and progression-free and overall survival following open versus robotic radical cystectomy: analysis from the RAZOR trial with a 3-year followup. J Urol 203(3):522–529. 10.1097/JU.0000000000000565 31549935 10.1097/JU.0000000000000565 PMC7487279

[19] Nix J, Smith A, Kurpad R, Nielsen ME, Wallen EM, Pruthi RS (2010) Prospective randomized controlled trial of robotic versus open radical cystectomy for bladder cancer: perioperative and pathologic results. Eur Urol 57(2):196–201. 10.1016/j.eururo.2009.10.024 19853987 10.1016/j.eururo.2009.10.024

[20] Styn NR, Montgomery JS, Wood DP et al (2012) Matched comparison of robotic-assisted and open radical cystectomy. Urology 79(6):1303–1308. 10.1016/j.urology.2012.01.055 22516354 10.1016/j.urology.2012.01.055

[21] Sathianathen NJ, Pan HYC, Furrer M et al (2023) Comparison of robotic vs open cystectomy: a systematic review. Bladder Cancer 9(3):253–269. 10.3233/BLC-220065 38993188 10.3233/BLC-220065 PMC11181804

[22] Nepple KG, Strope SA, Grubb RL 3rd, Kibel AS (2013) Early oncologic outcomes of robotic vs. open radical cystectomy for urothelial cancer. Urol Oncol 31(6):894–898. 10.1016/j.urolonc.2011.06.009 21803615 10.1016/j.urolonc.2011.06.009 PMC4090045

[23] Kassouf W, Agarwal PK, Grossman HB et al (2009) Outcome of patients with bladder cancer with pN+ disease after preoperative chemotherapy and radical cystectomy. Urology 73(1):147–152. 10.1016/j.urology.2008.07.035 18848348 10.1016/j.urology.2008.07.035 PMC2674246

[24] Mertens LS, Meijer RP, Meinhardt W et al (2014) Occult lymph node metastases in patients with carcinoma invading bladder muscle: incidence after neoadjuvant chemotherapy and cystectomy vs after cystectomy alone. BJU Int 114(1):67–74. 10.1111/bju.12447 24053889 10.1111/bju.12447

[25] Jeong H, Park KJ, Lee Y et al (2022) The prognosis and the role of adjuvant chemotherapy for node-positive bladder cancer treated with neoadjuvant chemotherapy followed by surgery. Cancer Res Treat 54(1):226–233. 10.4143/crt.2021.365 33957019 10.4143/crt.2021.365 PMC8756114

[26] Galsky MD, Hahn NM, Rosenberg J et al (2011) Treatment of patients with metastatic urothelial cancer “unfit” for Cisplatin-based chemotherapy. J Clin Oncol 29(17):2432–2438. 10.1200/JCO.2011.34.8433 21555688 10.1200/JCO.2011.34.8433

[27] Bajorin DF, Witjes JA, Gschwend JE et al (2021) Adjuvant nivolumab versus placebo in muscle-invasive urothelial carcinoma. N Engl J Med 384(22):2102–2114. 10.1056/NEJMoa2034442 34077643 10.1056/NEJMoa2034442 PMC8215888

